# Isolated Duodenal Crohn's Disease: A Case Report and a Review of the Surgical Management

**DOI:** 10.1155/2013/421961

**Published:** 2013-05-23

**Authors:** Faruk Karateke, Ebru Menekşe, Koray Das, Sefa Ozyazici, Pelin Demirtürk

**Affiliations:** ^1^Numune Training and Research Hospital, Department of General Surgery, 01170 Adana, Turkey; ^2^Numune Training and Research Hospital, Department of Pathology, 01170 Adana, Turkey

## Abstract

Crohn's disease may affect any segment of the gastrointestinal tract; however, isolated duodenal involvement is rather rare. It still remains a complex clinical entity with a controversial management of the disease. Initially, patients with duodenal Crohn' s disease (DCD) are managed with a combination of antiacid and immunosuppressive therapy. However, medical treatment fails in the majority of DCD patients, and surgical intervention is required in case of complicated disease. Options for surgical management of complicated DCD include bypass, resection, or stricturoplasty procedures. In this paper, we reported a 33-year-old male patient, who was diagnosed with isolated duodenal Crohn's diseases, and reviewed the surgical options in the literature.

## 1. Introduction

Duodenal Crohn's disease (DCD) has been reported to occur in 0.5% to 4% of patients with Crohn's disease [1]. The first report of duodenal involvement was described by Gottlieb and Alpert in 1937 [1–3]. Since then, DCD still remains a complex clinical entity with a controversial management of the disease. The most common site of duodenal Crohn's disease is the duodenal bulb, and obstruction is the most frequent presentation [1, 4]. Medical management with antiinflammatory and antiacid medications is effective in patients without obstruction. However, surgery has been reported to be necessary for as many as 91% of patients with obstruction [1, 5, 6]. Options for surgical management of complicated DCD include bypass, resection, or stricturoplasty. Resection has been abandoned because of associated increased morbidity; therefore, bypass procedures and stricturoplasty have become the accepted surgical options for DCD [5, 7–9]. Although Crohn's disease can involve any segment of the gastrointestinal tract, isolated Crohn's disease of duodenum without extraduodenal involvement is extremely rare. In this report, we described an isolated case of DCD and reviewed the surgical options.

## 2. Case

**Figure 1:**
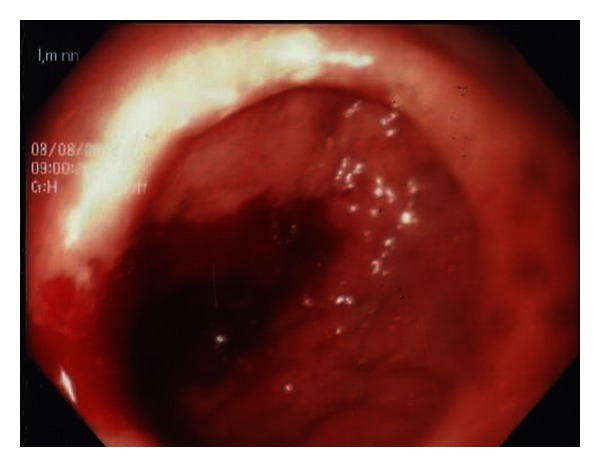
Esophagogastroduodenoscopy findings of the patient: a tight stricture with mucosal edema and the longitudinal ulcerations in the duodenal bulb with a near-complete obstruction.

**Figure 2:**
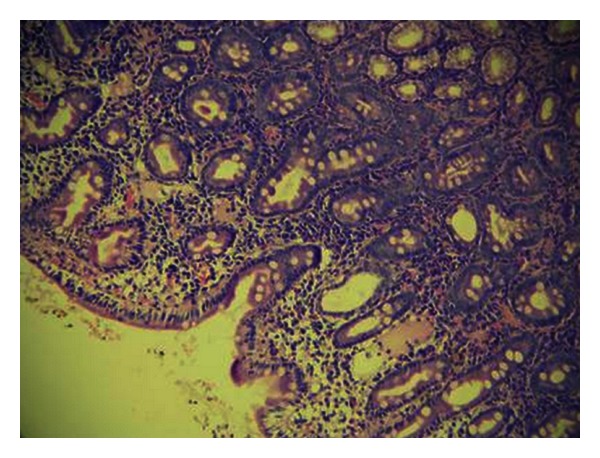
Foci of villous blunting, glandular destruction, mixed chronic inflammatory infiltrate in lamina propria, and cryptitis (H&Ex200).

**Figure 3:**
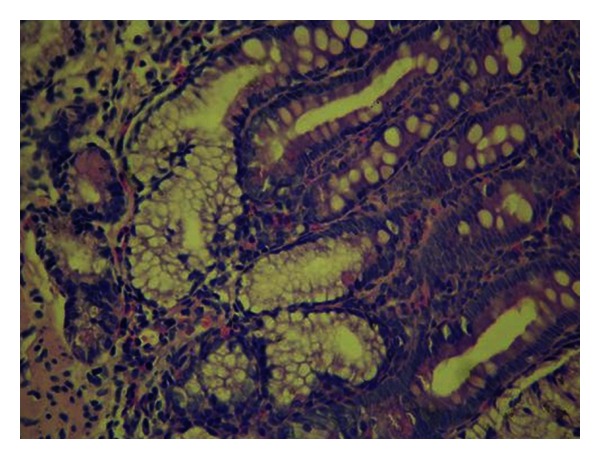
Pyloric metaplasia at the base of the crypt (H&Ex400).

A 33-year-old male patient was referred to our clinic with a 6-month history of intermittent, abdominal pain accompanied by progressive nausea, bilious emesis, and weight loss. His defecation habits were normal. On physical examinations, only a slight tenderness and fullness was noted in the epigastric region. Routine blood work revealed a mild normocytic anemia (Hgb: 12,0 g/dL, normal range: 13,5–17,2 g/dL). Biochemical parameters were unremarkable. He subsequently underwent an esophagogastroduodenoscopy (EGD), abdominal computerized tomography (CT), and colonoscopy. EGD revealed a tight stricture with mucosal edema and the longitudinal ulcerations in the duodenal bulb with a near-complete obstruction (Figure 1). The biopsy specimens of the duodenum showed severe inflammation, mixed chronic inflammatory infiltrate in lamina propria, and cryptitis with the evidence of DCD (Figures 2 and 3). CT and colonoscopy were normal. Based on these clinical, radiological, and pathological findings, isolated DCD was diagnosed, and total parenteral nutrition therapy was initiated along with nasogastric decompression. After having the nutritional status of the patient improved, he went on laparoscopic exploration. A stricture was found in the first part of the duodenum with a dilated stomach. A laparoscopic gastrojejunostomy was performed without vagotomy. The patient tolerated the procedure well and was discharged without any adverse event on postoperative 7th day, and thereafter, he was referred to the gastroenterology department for adjuvant therapy. He was noted to be on remission without any complaints during a 9-month followup under proton-pump inhibitors treatment.

## 3. Discussion

Crohn's disease is a chronic and inflammatory disease characterized by the segmented, transmural involvement of the alimentary tract that can affect any part of the system from the mouth to the anus [10]. Patients with DCD usually present with Crohn's disease affecting other areas of the gastrointestinal tract; however, isolated DCD is a very rare clinical entity [1, 4]. Initially, patients with DCD are managed with a combination of antiacid and immunosuppressive therapy. However, medical treatment fails in the majority of DCD patients, and surgical intervention is required in case of complicated disease. The most common indication for surgical intervention is progressive obstruction, failure of medical management with intractable pain, bleeding, perforation, and fistulous disease [1, 5, 6].

Options for surgical treatment of complicated DCD disease include resection, bypass, or strictureplasty. Resection procedure that was described by Allen Whipple has been associated with significant morbidity and mortality. Short gut syndrome, diarrhea, chronic malnutrition, electrolyte derangements, vitamin deficiencies, and chronic anemia are the complications of resection [5, 11].

Because of high rates of morbidity and mortality, bypass procedures and strictureplasty have been considered as standard surgical options to preserve the duodenum and prevent related complications of surgical resection.

Strictureplasty was introduced by Lee and Papiaoannu in the 1970s and furthermore popularized by Alexander-Williams [12, 13]. The most common strictureplasty techniques for DCD are the Heineke-Mikulicz procedure for shorter strictures and the Finney strictureplasty for longer segments of disease [11]. In the early 1990s, strictureplasty techniques were preferred constantly over bypass procedures due to reliability, safety, and efficacy with their own pitfalls. However, due to the complexity of the strictureplasty arising from the retroperitoneal location of the duodenum and need for extensive mobilization of the duodenum, bypass procedures have been preferred recently.

Although bypass procedures are more technically feasible and safe compared to strictureplasty, they are associated more frequently with blind-loop syndrome, dumping, bile reflux gastritis, and marginal ulceration [9]. Only a limited number of studies comparing outcomes after strictureplasty and bypass surgery for duodenal disease are available in the recent literature. The results of these studies have been controversial. Worsey et al. performed strictureplasty for 13 patients and bypass procedures for 21 patients. They concluded that duodenal strictureplasty is safe and effective and may in fact have potential anatomic and physiologic advantages over bypass procedures [9].

Yamamoto et al. performed duodenal strictureplasty for 13 patients, 9 of which required further surgical intervention due to early postoperative complications and restricturing at the strictureplasty site (*n* = 6) in a median followup of 143 months. Similarly, 13 patients underwent bypass procedures. In this cases, no patients required reoperation for early postoperative complications; however, 6 patients required further surgical intervention at a later date (median followup 192 months) for stomal ulceration (*n* = 2) and anastomotic obstruction (*n* = 4) [8]. 

Yamamoto et al. stated that strictureplasty had no patent advantages over bypass and was associated with a higher incidence of early complications and restricturing [8].

One of the largest study published on the surgical management of patients with DCD was conducted by Shapiro et al., which was the first to present the laparoscopic experience in bypass procedures [3]. In this study, thirty patients had surgical intervention for DCD, including 4 patients with isolated disease. The surgical procedures were open bypass for 11 patients, laparoscopic bypass for 13, and stricturoplasty for 2 patients. Early complications rate was 8% in laparoscopic procedures and 36% in open bypass procedures, respectively. This lower complication rate was attributed to a decrease in complication rate after laparoscopic surgery. Also Shapiro et al. revealed that there is no role for vagotomy in bypass procedures to prevent marginal ulceration in the era of wide use of proton pump inhibitors (PPI). Adjuvant treatment options after surgery of isolated DCD include PPI, H2 receptor antagonists, sucralfate, or steroids.

The clinical presentation of our patient was progressive obstruction with symptoms of isolated duodenal involvement as his first clinical manifestation of the disease. Based on the previous studies, we performed laparoscopic gastrojejunal bypass for surgical intervention, and he was noted to be on remission after surgery during 9 months under PPI therapy.

## 4. Conclusion

The optimal management of duodenal Crohn's disease should be individualized on a case-by-case basis. Laparoscopic gastrojejunal bypass can be an alternative in the treatment of DCD.
